# A Multi-Center Competing Risks Model and Its Absolute Risk Calculation Approach

**DOI:** 10.3390/ijerph16183435

**Published:** 2019-09-16

**Authors:** Jintao Wang, Zhongshang Yuan, Yi Liu, Fuzhong Xue

**Affiliations:** 1 Department of Biostatistics, School of Public Health, Shandong University, Jinan 250012, China; wangjintao0214@sdu.edu.cn (J.W.); yuanzhongshang@sdu.edu.cn (Z.Y.); liuyi238@sdu.edu.cn (Y.L.); 2 Department of Statistics, School of Mathematics and Statistics, Shandong University, Weihai 264209, China

**Keywords:** absolute risk, area under the curve, competing risk, multi-center, risk assessment

## Abstract

In the competing risks frame, the cause-specific hazard model (CSHM) can be used to test the effects of some covariates on one particular cause of failure. Sometimes, however, the observed covariates cannot explain the large proportion of variation in the time-to-event data coming from different areas such as in a multi-center clinical trial or a multi-center cohort study. In this study, a multi-center competing risks model (MCCRM) is proposed to deal with multi-center survival data, then this model is compared with the CSHM by simulation. A center parameter is set in the MCCRM to solve the spatial heterogeneity problem caused by the latent factors, hence eliminating the need to develop different models for each area. Additionally, the effects of the exposure factors in the MCCRM are kept consistent for each individual, regardless of the area they inhabit. Therefore, the coefficient of the MCCRM model can be easily explained using the scenario of each model for each area. Moreover, the calculating approach of the absolute risk is given. Based on a simulation study, we show that the estimate of coefficients of the MCCRM is unbiased and precise, and the area under the curve (AUC) is larger than that of the CSHM when the heterogeneity cannot be ignored. Furthermore, the disparity of the AUC increases progressively as the standard deviation of the center parameter (SDCP) rises. In order to test the calibration, the expected number (E) of strokes is calculated and then compared with the corresponding observed number (O). The result is promising, so the SDCP can be used to select the most appropriate model. When the SDCP is less than 0.1, the performance of the MCCRM and CSHM is analogous, but when the SDCP is equal to or greater than 0.1, the performance of the MCCRM is significantly superior to the CSHM. This suggests that the MCCRM should be selected as the appropriate model.

## 1. Introduction

Cardio-cerebrovascular diseases have become a severe public health issue. In particular, stroke is the primary cause of disability. With the aging population, the ante-displacement of the age of disease onset, and the improvement of the material standard of living, the prevention and treatment of stroke is still a great challenge worldwide [1,2]; therefore, the development of new theories and methods is of utmost importance [3,4]. Carrying out risk assessments on individuals prior to stroke can provide important information for medical research, hence reduce the social economic burden in the future. To this end, either the effects of multiple covariates for each individual must be determined, or the absolute risk of every person can be calculated through regression or other approaches. The most commonly used regression analysis for risk assessment is the Cox model [5]. The traditional hazard-based Cox model uses a semi-parametric setting with non-parametric baseline hazard, perfect link and exponential functions. However, although it has been widely used in medical studies, cox model ignores the existence of competing risks.

Medical practice produce a large amount of competing risks data, which is especially related to elderly people [6,7]. A common type of competing risks data is survival data with multiple causes of death. For example, in a clinical trial that compares different treatment therapies for breast cancer, interest may be focused on death from breast cancer, but a patient may die due to causes other than breast cancer, such as coronary heart disease, hospital infection, or a traffic accident [8]. The standard methods for analyzing competing risks data include cause-specific hazard functions [9,10], subdistribution hazard model [11], Framingham models [12,13,14] and fine adjustment Framingham models, depending on the population characteristics. However, the application and imitation of Framingham models have caused many problems, such as variables significant in clinical treatment becoming insignificant in models or coefficients being unexplainable [15,16,17,18]. Therefore, a more adequate modeling approach is needed for the stroke patients in China.

Sometimes, the observed covariates cannot explain the large proportion of variation in time-to-event data from different areas, for instance, the data of multi-center clinical trials or multi-center cohort studies [19,20,21,22,23]. China has a vast territory and many ethnic groups. The heterogeneity of the public in different areas caused by climate, economic level, living habits, and many other factors, is enormous. Therefore, the effects of these latent factors should not be ignored when studying the risk factors of diseases and calculating the absolute risk, even though some of these important factors are unavailable in some circumstances. Furthermore, the effect of a specified covariate should be consistent for individuals at different centers, according to the risk assessment in survival analysis. Therefore, in a multi-center competing risks scenario, with the presence of heterogeneity caused by some latent factors, it is inappropriate to establish different models for every center, even the sample size at each center is sufficient.

In this paper, we demonstrate that most of the predictors (covariates) are not effected by spatial heterogeneity. For example, for smokers with the same amount of cigarette consumption each day, smoking should have the same effect in different areas. That is, the effect of smoke is not related to the smoker’s geographical location. However, it is not ideal if we established different models for different areas, because we may obtain different odds ratios about smoke. Therefore, for multi-center cohort data or multi-center randomized controlled trials (RCT), we established a uniform model for different areas based on the Frailty model [24,25,26,27], while also setting a center parameter to eliminate the problem detailed above. All the effects of latent factors were incorporated in the center parameter, which was helpful for obtaining accurate and consistent estimators of all of the predictors. Furthermore, based on the Gail model [28], we estimated the absolute risk of stroke for each person under the competing risks frame. These methods can be easily applied to other cardio-cerebrovascular diseases, and perhaps even to other diseases.

The rest of this paper is organized as follows. Section 2 reviews the cause-specific hazard model (CSHM), and then introduces the multi-center competing risks model (MCCRM) and the approach of calculating absolute risks. Section 3 presents the results of the simulation study, assesses the performance of the proposed model, and describes the calibration of the approach to calculate absolute risks. Section 4 compares the performance of the two models (CSHM and MCCRM) on a dataset from the Shandong Center for Disease Control and Prevention. Section 5 presents the discussion. Concluding remarks are given in Section 6.

## 2. Methods

### 2.1. The Cause-Specific Hazard Model

For the CSHM, without loss of generality, we assumed that there were only two causes of failure for the following description [9,10]. For example, when death caused by stroke was the event of interest, all other causes of death were treated as competing risks. The cause-specific hazard function is

$$\alpha (t|Z(t))=\underset{\Delta t\to 0}{\lim}P\{t\leq T<t+\Delta t|T\geq t;Z(t)\}/\Delta t$$

where Z(t) denotes the value of the regression vector at time t [5]. If cases with only two competing risks, the proportional cause-specific hazard model based on the Cox model can be expressed as

$$\alpha_{01}(t|Z)=\alpha_{01;0}(t)\exp(\beta_{01}^{T}Z)$$

$$\alpha_{02}(t|Z)=\alpha_{02;0}(t)\exp(\beta_{02}^{T}Z).$$

### 2.2. The Multi-Center Competing Risks Model

This model deals with a multi-center scenario with the presence of heterogeneity. $Z_{ki}(k=1,2,\cdots ,K;i=1,2,\cdots ,n_{k})$ denotes the covariates vector, where *K* is the number of centers, and *n_k_* is the number of individuals in the *k*th center. We add a center parameter $\eta_{k}$ to the CSHM, so the MCCRM can be given as

(1) $$\alpha_{01}^{\left(k\right)}(t|Z_{ki})=\alpha_{01;0}(t)\exp(\beta_{01}^{T}Z_{ki}+\eta_{k})$$

(2) $$\alpha_{02}^{\left(k\right)}(t|Z_{ki})=\alpha_{02;0}(t)\exp(\beta_{02}^{T}Z_{ki}+\eta_{k})$$

The center parameter $\eta_{k}$ incorporates all other effects of latent factors (covariates) such as economy, politics, climate, and living habits. $\alpha_{01;0}(t)$ and $\alpha_{02;0}(t)$ are the baseline hazard functions. The coefficients vector $\beta_{0j}$ is consistent for every center. This means that the effect of the specified covariate given to other covariates to every individual is consistent. For example, smoke has the same negative effect for each person regardless of the center or area they belong to.

Under the multi-center competing risks scenario, we can derive the following formulas according to reference [7].

The all causes hazard can be expressed as

(3) $$\alpha_{0}^{\left(k\right)}(t|Z_{ki})=\alpha_{01}^{\left(k\right)}(t|Z_{ki})+\alpha_{02}^{\left(k\right)}(t|Z_{ki})$$

and the cumulative hazard for the *k*th is

(4) $$A_{0}^{\left(k\right)}(t|Z_{ki})=\int_{0}^{t}\alpha_{0}^{\left(k\right)}(u|Z_{ki})du.$$

Therefore, the distribution function for the *i*th individual who belongs to the *k*th center is

(5) $$\begin{array}{c} F^{\left(k\right)}(t|Z_{ki})=P(T\leq t|Z_{ki})=1-\exp\left\{-A_{0}^{\left(k\right)}(t|Z_{ki})\right\}\\ =1-\exp\left\{-\int_{0}^{t}\left[\alpha_{01;0}(u)\exp(\beta_{01}^{T}Z_{ki}+\eta_{k})+\alpha_{02;0}(u)\exp(\beta_{02}^{T}Z_{ki}+\eta_{k})\right]du\right\}. \end{array}$$

From here, we assumed that our data had been stratified by gender and age; in other words, the MCCRM was developed using data from individuals with the same gender and age period. In such a scenario, the baseline hazard $\alpha_{01;0}(u)$ and $\alpha_{02;0}(u)$ can be assumed to be constant, and Equation (5) can be simplified as follows:

(6) $$F^{\left(k\right)}(t|Z_{ki})=1-e^{-\lambda t}$$

where $\lambda =\alpha_{01;0}(u)\exp(\beta_{01}^{T}Z_{ki}+\eta_{k})+\alpha_{02;0}(u)\exp(\beta_{02}^{T}Z_{ki}+\eta_{k})$.

As mentioned above, the distribution function of the event of interest for the *i*th individual who belongs to the *k*th center has a similar expression to Equation (6):

(7) $$F_{01}^{\left(k\right)}(t|Z_{ki})=1-e^{-\lambda_{01}t}$$

where $\lambda_{01}=\alpha_{01;0}(u)\exp(\beta_{01}^{T}Z_{ki}+\eta_{k})$.

Therefore,

(8) $$\alpha_{01;0}(u)\exp(\beta_{01}^{T}Z_{ki}+\eta_{k})=-\frac{1}{t}\ln(1-F_{01}^{\left(k\right)}(t|Z_{ki})).$$

At the baseline level, we assumed that all of the covariates and the center parameter equaled to zero; thus Equation (8) was simplified as

(9) $$\alpha_{01;0}(u)=-\ln(1-P)$$

where P is the overall incidence of all centers.

$P_{k}$ was taken as the incidence of the kth center, so the center parameter $\eta_{k}$ could be calculated as follows:

(10) $$\eta_{k}=\ln(-\ln(1-P_{k}))-\ln(-\ln(1-P)).$$

A detailed derivation of the formula can be found in Appendix A.

Now, we give the absolute risk equation as in the Gail model [23]:

(11) $$P\{a,\tau |Z_{ki}\}=\int_{a}^{a+\tau }l_{k}\alpha_{01;0}(t)r(t)\exp\left\{-\int_{a}^{t}l_{k}\alpha_{01;0}(u)r(u)du\right\}\frac{S_{2}(t)}{S_{2}(a)}dt$$

where a is the age of the *i*th individual of the *k*th center and τ is a time interval. $P\{a,\tau |Z_{ki}\}$ is the absolute risk that a person has a certain disease in the time interval [a,a+τ] with covariates $Z_{ki}$. The relative risk r(t) is calculated as follows:

(12) $$r(t)=\mathrm{lk}\cdot \exp(\beta_{01;a}^{T}\cdot {\tilde{Z}}_{ki})=e^{\eta_{k}}\cdot \exp(\beta_{01;a}^{T}\cdot {\tilde{Z}}_{ki}).$$

In addition, in Equation (11), $S_{2}(t)=\exp\left\{-\int_{0}^{t}l_{k}\alpha_{02;0}(u)du\right\}$.

As our model was established after the data had been stratified by age and gender, the baseline hazard could be set as constant. Therefore, Equation (11) was simplified as follows:

(13) $$P\{a,\tau |Z_{ki}\}=\frac{\xi \exp\left\{\xi \cdot a\right\}\cdot \left[\exp\lambda (a+\tau )-\exp(\lambda a)\right]}{S_{2}(a)\cdot \lambda }$$

where $\xi =l_{k}\alpha_{01;0}(t)r(t)$ and $\lambda =-\xi -l_{k}\alpha_{02;0}(t)$.

## 3. Simulations

In this section, we show the performances of the CSHM and the MCCRM. First, we generated random data with the method introduced by Jan Beyersmann et al. [7,29] and then established the two models with random data. Next, we assessed the above-mentioned models through statistical parameters such as bias, standard deviation (SD), root mean square error (RMSE), and area under the curve (AUC). Finally, we calibrated the multi-center competing risks model (MCCRM) by calculating the ratio of the expected number (E) of strokes in the given time interval and compared it with the corresponding observed number (O), i.e., E/O.

In the simulation, we chose stroke as the dependent variable. Death from stroke was the outcome of interest, and death from other causes was the competing risk. Using the Framingham models [12,13,15,30], we chose five factors as covariates: Total cholesterol (TC), high density lipoprotein (HDL), systolic blood pressure (SBP), diabetes, and smoking. Age and gender were used as stratified variables.

### 3.1. The Generation of the Dataset

Firstly, we generated the random data of covariates according to the real data used in the study by Zhenxin Zhu et al. [31]. The real data came from a cohort of all participants who received routine health check-ups from 2005 to 2010 at the Center for Health Management of Shandong Provincial QianFoShan Hospital and the Health Examination Center of Shandong Provincial Hospital. For TC, HDL and SBP were continuous variables, and we calculated the mean vector and covariance matrix of the three covariates using the real data. Then, random data were generated with a multivariate normal distribution. Diabetes and smoke were variables with values of 0–1, which were generated by binomial distribution with the rate of real data from Shouguang City, Shandong Province, China. The center parameter was calculated using Equation (10).

Secondly, due to the existence of competing risks, we generated random data from the dependent variables, which were survival time and survival outcome. As we stratified the data by age and gender, the baseline hazards in Equation (6) could be set as constants, and the covariates could be treated as approximately time-independent; therefore, the distribution of survival time could be simplified as an exponential distribution for each person. The true values of coefficients of the selected five covariates (exposure factors) in Equation (6) were set as $\beta_{01}=(1,-3,0.01,1,1)$ according to a rough estimate by the Cox model with real data. As the influence of covariates for other competing risks is always regarded as insignificant, the true values of coefficients of covariates for competing risks in Equation (6) were set as $\beta_{02}=(0.01,-0.01,0.0001,0.01,0.01)$.

At this point, we had obtained the survival time *T*. Next, we generated the survival outcome *X*. There were three outcomes, which were indicated by 0, 1, and 2, where 0 referred to a censored outcome, 1 indicated death from stroke, and 2 indicated death from causes other than stroke. We used $\alpha_{01}(t)/\left[\alpha_{01}(t)+\alpha_{02}(t)\right]$ as the parameter of binomial distribution to generate the outcome (1 or 2) for each sample. We generated random data *C* with a uniform distribution [0, b], and then specified 0 for the sample if *C* was smaller than *T* for each person, or 1 otherwise. The right endpoint *b* was used to control the censored ratio.

### 3.2. The Assessment of Models

Then, we were able to establish the CSHM and the MCCRM with the random data generated above. We used the packages survival, pROC, and MASS in R software [32] to conduct the analysis [33,34,35,36]. The total sample sizes of the simulated data were N = 1000 and 5000, with censored ratios of Q = 0.2 and 0.4. For each combination of N and Q, we set the standard deviation of the center parameter SDCP to 0.01, 0.05, 0.1, 0.5, 1.0, 1.5, and 2.0, respectively. The cycle time was specified as 1000 for every combination of N, Q, and SDCP. Then, we calculated the means of the bias, SD, RMSE, and AUC. Here, we give three examples (Table 1, Table 2 and Table 3) of combinations of N, Q, and SDCP. The parameter vectors of the N, Q, and SDCP are 5000, 0.2, and 0.01, respectively, for the values given in Table 1; 5000, 0.2, and 1.0 for the values given in Table 2; and 5000, 0.2, and 2.0 for the values given in Table 3.

**Figure 1:**
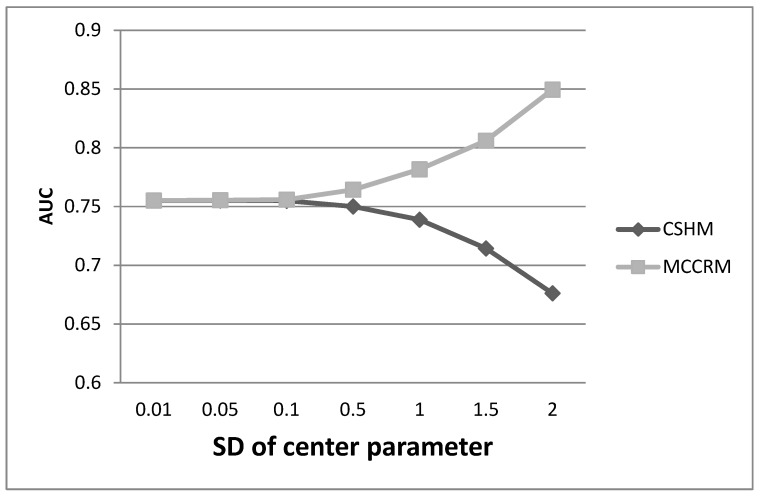
Comparison of the MCCRM and CSHM. Seven discrete points of standard deviation of the center parameter were specified for the simulations. AUC: area under the curve.

Table 1 shows that there was no significant difference between the performance of the MCCRM and the CSHM when the SDCP was equal to 0.01. However, when the SDCP was equal to 1.0 or 2.0, the estimate of coefficients of the MCCRM were more precise than those of the CSHM (Table 2 and Table 3). Additionally, the AUC of the MCCRM was significantly greater than that of the CSHM. According to the simulation results, the estimators of coefficients of MCCRM can be seen as unbiased compared with the true value $\beta_{01}=(1,-3,0.01,1,1)$. Furthermore, through a large number of simulations, we found that when the SDCP was less than 0.1, there was no significant difference between the two models. When the SDCP was greater than 0.1, the AUC of the MCCRM was significantly greater than that of the CSHM, and the difference increased progressively with the increase in the SDCP (Figure 1). When the SDCP was greater than 0.1, the estimate of coefficients of the MCCRM was more precise than those of the CSHM, and the difference was highly significant.

No obvious difference in performance was shown between censor ratios of Q = 0.4 and Q = 0.2. Further the performance was similar with sample sizes of N = 1000 and N = 5000. The robustness of the estimators (bias, SD, RMSE, AUC, E/O, etc.) was not good when N was less than 1000, and the performance of the statistics was robust when N was sufficient.

### 3.3. The Calibration of Models

Table 4 presents the calibration of the MCCRM. The field name t-i expresses the time interval [0,i],i=1,2,⋯,5. SDCP is the standard deviation of the center parameter, and only the simulation results of SDCPs equal to 0.5 or 1.0 are listed. The value of the first cell, 1.0510, represents the E/O at the given time interval [0,1]. E is the expected number, which is the sum of the absolute risk of every sample generated in Section 3.1. O is the observed number, which is the number of samples whose observed time was less than or equal to 1, and the observed cause was 1 (the outcome of interest).

Table 4 shows that the E/O was acceptable when the SDCP was equal to 0.5. However, when the SDCP was equal to 1.0 and the time length was greater than 3, the E/O was unsatisfactory. Through many simulations, we found that the E/O was acceptable when the SDCP was less than or equal to 0.5 and the time length was less than or equal to 5. Additionally, the precision of E/O decreased linearly with an increase in SDCP.

## 4. Illustration

We obtained data from the Shandong Center for Disease Control and Prevention study from patients with four diseases (stroke, coronary heart disease (CHD), lung cancer, and stomach cancer) from 17 cities in Shandong Province, China in 2015. For every disease, we had data on the incidence number and population size of the 17 cities, which were stratified by age (five years for each interval). Furthermore, lung cancer and stomach cancer were stratified by gender. We calculated the incidence for each city and then calculated the SDCP after the transformation of incidence using Equation (10). Table 5 shows SDCP for the four diseases of patients whose age was equal or greater than 40. According to the results in Section 3, when the SDCP is greater than 0.1, the heterogeneity across different centers cannot be ignored. From Table 5, we can see that all of the numbers were significantly greater than 0.1; thus, it is necessary to emphasize the importance of the MCCRM during the practical application of multi-center data.

We chose stroke to illustrate the performance of the MCCRM. The results of other diseases were analogous. Table 6 presents the comparisons of the regression coefficients and the AUC of the two models with stroke. We used the stroke incidence of the 17 cities to generate the center parameter with Equation (10), and the age interval was equal or greater than 50 and less than 55. The covariates survival time and survival outcome were generated by the same method introduced in detail in Section 3. The sample size was 5000, and the censored ratio was 0.2. The SDCP 1.047 of ages 50 to 55, which according to Table 5, is obviously greater than 0.1.

As the results of the simulation in Section 3, Table 6 shows that all of the estimators (bias, SD, RMSE) of the MCCRM were more precise and superior to the corresponding estimators of the CSHM. For example, the maximum of RMSE of the CSHM was 0.8580, while the RMSE of the same covariate (HDL) in the MCCRM was 0. 079. The AUC of the MCCRM was 0.7994, while the AUC of the CSHM was only 0.7205.

## 5. Discussion

A common question arising in multi-center random clinical trials and multi-center cohort studies where competing risks exist is whether any heterogeneity in outcomes exists, and whether the heterogeneity has an obvious influence on the research target. Therefore, it is necessary to choose the appropriate model and determine whether statistical adjustment is required while estimating the effect of risk factors or calculating the absolute risk of a certain disease. When analyzing multi-center survival data, frailty survival models have been shown being useful, notably with regard to the usual large number of centers and low number of patients in each center [37,38]. Nevertheless, frailty survival models do not provide any detailed differences of the CSHM and frailty models. Through the simulation in Section 3, we have provided a precise analysis of the CSHM and the MCCRM by changing the sample size, censored ratio, the standard deviation of the center parameter, and the number of centers, among other factors. Theoretical studies will be presented in follow-up work.

With existing competing risks, Bayesian statistics have been reported to be more useful and efficient for assessing prior information, variable selection, and absolute risk [39,40,41,42]. Moreover, Bayesian models are more flexible than empirical models. However, in this paper, in order to emphasize the importance of heterogeneity, we have only mentioned the multi-center data, and did not take prior information into account. The limitation of this paper is that only baseline data are used for prediction, and the situation of multiple follow-up observations is not fully considered. This will inevitably affect the accuracy of predicting absolute risk and the stability of the model. In our follow-up work, we will consider combining the multiple follow-up observations, the prior information and multi-center data under a competing risks scenario.

## 6. Conclusions

Through Equation (10), we calculated the SDCP, which helped us select the most appropriate model according to the physical truth. When the SDCP was less than 0.1, the MCCRM and CSHM performed analogously, so either could be selected randomly for the practical application. When the SDCP was equal to or greater than 0.1, the performance of the MCCRM was significantly superior to the CSHM according to estimators such as bias, SD, RMSE, AUC, and E/O. Furthermore, when the SDCP was too big, the CSHM became inefficient, then the MCCRM should be selected as the appropriate model. Therefore, MCCRM can help us make full use of multi-center data and give accurate estimates of covariate coefficients. Moreover, the covariate coefficients of the MCCRM are consistent for different centers or areas, so the explanation of covariate coefficients has become more simple and reasonable.

Using Equation (11) and the MCCRM, the absolute risk of stroke occurring for a certain person was calculated. The approach of calculating absolute risk was excellent when the time interval was not too large, according to the calibration in Section 3. That is, short time intervals were predicted more precisely than long time intervals. However, only having the baseline value of covariates in a cohort study may cause inaccuracy in long time interval prediction.

## Figures and Tables

**Table 1:** Comparison of the coefficients of the two models.

Covariate	True Value	CSHM (AUC = 0.755)			MCCRM (AUC = 0.755)		
		Bias	SD	RMSE	Bias	SD	RMSE
TC		0.0001	0.0201	0.0200	0.0002	0.0201	0.0201
HDL	−3	0.0003	0.0771	0.0771	0.0000	0.0771	0.0771
SBP	0.01	0.0000	0.0008	0.0008	0.0000	0.0008	0.0008
Diabetes	1	0.0008	0.0527	0.0527	0.0009	0.0527	0.0527
Smoking	1	−0.0001	0.0303	0.0303	0.0000	0.0303	0.0303

Note: Sample size: 5000; censor ratio: 0.2; standard deviation of the center parameter: 0.01. CSHM: cause-specific hazard model, HDL: high-density lipoprotein; MCCRM: multi-center competing risks model; SBP: systolic blood pressure; TC: total cholesterol; SD: standard deviation.

**Table 2:** Comparisons of the coefficients of the two models.

Covariate	True Value	CSHM (AUC = 0.7388)			MCCRM (AUC = 0.7817)		
		Bias	SD	RMSE	Bias	SD	RMSE
TC	1	−0.2392	0.0196	0.2400	0.0015	0.0201	0.0201
HDL	−3	0.7181	0.0776	0.7223	−0.0060	0.0767	0.0769
SBP	0.01	−0.0024	0.0008	0.0025	0.0000	0.0007	0.0007
Diabetes	1	−0.2365	0.0576	0.2434	0.0039	0.0557	0.0559
Smoking	1	−0.2400	0.0309	0.2420	0.0005	0.0317	0.0317

Note: Sample size: 5000; censor ratio: 0.2; standard deviation of the center parameter: 1.0.

**Table 3:** Comparisons of coefficients of the two models.

Covariate	True Value	CSHM (AUC = 0.6762)			MCCRM (AUC = 0.8495)		
		Bias	SD	RMSE	Bias	SD	RMSE
TC	1	−0.5444	0.0192	0.5447	0.0004	0.0202	0.0202
HDL	−3	1.6292	0.0739	1.6309	−0.0022	0.0776	0.0776
SBP	0.01	−0.0054	0.0008	0.0055	0.0000	0.0008	0.0008
Diabetes	1	−0.5568	0.0643	0.5605	−0.0015	0.0573	0.0573
Smoking	1	−0.5339	0.0296	0.5347	0.0010	0.0301	0.0301

Note: Sample size: 5000; censor ratio: 0.2; standard deviation of the center parameter: 2.0.

**Table 4:** The E/O (expected number/observed number) of the MCCRM.

SDCP	t-1	t-2	t-3	t-4	t-5
0.5	1.0510	1.0476	0.9630	1.0980	1.0688
1.0	1.0191	1.0566	1.1277	1.3810	1.9183

**Table 5:** The SDs of the center parameter of four diseases.

		40~	45~	50~	55~	60~	65~	70~	75~	80~	85~
Stroke		1.047	1.017	1.047	0.983	0.869	0.972	0.867	0.849	0.730	0.756
CHD		0.882	NA	0.624	0.731	0.716	0.822	0.726	0.696	0.663	0.750
Lung cancer	F	0.452	0.368	0.476	0.541	0.464	0.561	0.495	0.515	0.608	0.654
	M	0.410	0.430	0.547	0.510	0.499	0.526	0.520	0.563	0.550	0.582
Stomach cancer	F	0.481	0.433	0.512	0.556	0.365	0.457	0.589	0.482	0.631	0.825
	M	0.532	0.489	0.549	0.462	0.532	0.546	0.483	0.606	0.582	0.635

Note: F: female; M: male; Data came from 17 cities in Shandong Province, China. CHD: coronary heart disease.

**Table 6:** Comparisons of the two models of stroke.

Covariate	True Value	CSHM (AUC = 0.7205)			MCCRM (AUC = 0.7994)		
		Bias	SD	RMSE	Bias	SD	RMSE
TC	1	−0.2835	0.0251	0.2846	0.0032	0.0192	0.0195
HDL	−3	0.8531	0.0916	0.8580	−0.0094	0.0785	0.0790
SBP	0.01	−0.0028	0.0009	0.0030	0.0001	0.0008	0.0008
Diabetes	1	−0.2984	0.0792	0.3087	0.0043	0.0548	0.0549
Smoking	1	−0.2765	0.0321	0.2784	0.0016	0.0316	0.0317

Note: Sample size: 5000; censor ratio: 0.2; standard deviation of the center parameter: 1.047.

## Abbreviations

CSHM	Cause-specific hazard model
MCCRM	Multi-center competing risks model
AUC	Area under the curve
SDCP	Standard deviation of the center-parameter
E/O	Expected number/corresponding observed number
SD	Standard deviation
RMSE	Root mean square error
TC	Total cholesterol
HDL	High density lipoprotein
SBP	Systolic blood pressure
CHD	Coronary heart disease

## Appendix A

Let $Z_{ki}(k=1,2,\cdots ,K;i=1,2,\cdots ,n_{k})$ denote the covariates vector, where K is the number of centers and $n_{k}$ is the number of individuals in the kth center. $\alpha_{01;0}(t)$ and $\alpha_{02;0}(t)$ are the baseline hazard functions. The coefficients vector $\beta_{0j}$ is consistent for every center. Then, the distribution function of survival time T is as follows,

$$\begin{array}{l} F^{\left(k\right)}(t|Z_{ki})=P(T\leq t|Z_{ki})=1-\exp\left\{-A_{0}^{\left(k\right)}(t|Z_{ki})\right\}\\ =1-\exp\left\{-\int_{0}^{t}\alpha_{0}^{\left(k\right)}(u|Z_{ki})du\right\}=1-\exp\left\{-\int_{0}^{t}\left[\alpha_{01}^{\left(k\right)}(u|Z_{ki})+\alpha_{02}^{\left(k\right)}(u|Z_{ki})\right]du\right\}\\ =1-\exp\left\{-\int_{0}^{t}\left[\alpha_{01;0}(u)\exp(\beta_{01}^{T}Z_{ki}+\eta_{k})+\alpha_{02;0}(u)\exp(\beta_{02}^{T}Z_{ki}+\eta_{k})\right]du\right\} \end{array}$$

If the baseline hazard functions are set as constant, then

$$\begin{array}{l} F^{\left(k\right)}(t|Z_{ki})=1-e^{-\lambda t}\\ where,\lambda =\alpha_{01;0}(u)\exp(\beta_{01}^{T}Z_{ki}+\eta_{k})+\alpha_{02;0}(u)\exp(\beta_{02}^{T}Z_{ki}+\eta_{k})\\ =e^{\eta_{k}}\left[\alpha_{01;0}(u)\exp(\beta_{01}^{T}Z_{ki})+\alpha_{02;0}(u)\exp(\beta_{02}^{T}Z_{ki})\right] \end{array}$$

Analogously, the distribution function of survival time *t* for cause 1 is as follows,

$$\begin{array}{l} F_{01}^{\left(k\right)}(t|Z_{ki})=1-e^{-\lambda_{01}t}\\ \mathrm{here},\lambda_{01}=\alpha_{01;0}(u)\exp(\beta_{01}^{T}Z_{ki}+\eta_{k})\\ =e^{\eta_{k}}\left[\alpha_{01;0}(u)\exp(\beta_{01}^{T}Z_{ki})\right] \end{array}$$

Thus,

$$\begin{array}{l} \ln(1-F_{01}^{\left(k\right)}(t|Z_{ki}))=-\lambda_{01}t\\ \lambda_{01}=-\frac{1}{t}\ln(1-F_{01}^{\left(k\right)}(t|Z_{ki})),\\ \alpha_{01;0}(u)\exp(\beta_{01}^{T}Z_{ki}+\eta_{k})=-\frac{1}{t}\ln(1-F_{01}^{\left(k\right)}(t|Z_{ki}))\\ \alpha_{01;0}(u)=\frac{-\ln(1-F_{01}^{\left(k\right)}(t|Z_{ki}))}{t\cdot \exp(\beta_{01}^{T}Z_{ki}+\eta_{k})} \end{array}$$

Under the baseline level, the covariates vector is not significant, so the formula is $\eta_{k}=\ln(-\ln(1-P_{k}))-\ln(-\ln(1-P))$.
